# Gastric Polyps in Long-Term Proton Pump Inhibitor Use: Identification of Risks and Characteristics

**DOI:** 10.7759/cureus.62365

**Published:** 2024-06-14

**Authors:** Malek Michael Bouhairie, Racha Elseblani, Remi Lakis, Mahmoud Hallal

**Affiliations:** 1 Department of Gastroenterology and Hepatology, Faculty of Medicine, Lebanese University, Beirut, LBN; 2 Department of Reanimation and Anesthesiology, Faculty of Medicine, Lebanese University, Beirut, LBN; 3 Department of Gastroenterology and Hepatology, Al Zahraa Hospital University Medical Center, Beirut, LBN

**Keywords:** helicobacter pylori, prevalence, duration of ppi use, gastric polyps, chronic proton pump inhibitors use

## Abstract

Aim

Estimate the prevalence of gastric polyps linked to long-term use of proton pump inhibitor (PPI), determine the various risk factors that promote this association, and identify the characteristics associated with these polyps.

Methods

This prospective cross-sectional study was conducted on approximately 1000 patients presenting to the Gastroenterology Endoscopic Department for upper GI endoscopy at two hospital centers in Beirut, Lebanon, over a period of 12 months from September 2021 to September 2022. The demographic and clinical data of patients who had been taking PPIs for at least one month were collected via a questionnaire. All patients with a previous Helicobacter pylori (H. pylori) infection, presence of hypergastrinemia, or a personal/family history of gastric polyps were excluded from this study. Statistical analyses were performed using SPSS 20 software. Categorical variables were compared by Fisher’s exact test; p-values of less than 0.05 were considered statistically significant.

Results

The prevalence of gastric polyps linked to long-term PPI use was 30%. The minimum duration of daily PPI use required for the formation of polyps is around 24 months. The dosage did not play a significant role in increasing this prevalence. A significant correlation was found between chronic PPI use and factors such as sex, age range, duration, and type of PPI used. These polyps were predominantly found in females (with an OR of 2.9), increased with age, were mostly of the fundic gland type, and their size was proportionally linked to both the dosage and duration of daily PPI use. No cases of dysplasia within the fundic gland polyps (FGPs) were demonstrated in our study.

Conclusion

To date, there is no current data that prove an association between gastric cancer and PPI-induced FGPs. Additionally, the incidence of FGPs has increased with the widespread chronic use of PPIs. Therefore, attention should be drawn to the potential risk of dysplasia. Thus, the present study highlights the importance of limiting the prescription of PPIs to globally well-defined indications and determining the various risk factors that promote the association between gastric polyps and PPI use.

This abstract was recently presented as an E-poster at the ESGE Days 2024 Congress on April 25-27, 2024, in Berlin, Germany.

## Introduction

Proton pump inhibitors (PPIs) are among the most potent inhibitors of gastric acid secretion worldwide, used in the prevention of non-steroidal anti-inflammatory drugs (NSAIDs)-induced gastro-duodenal injuries, and in the treatment of acid-related disorders, such as gastro-esophageal reflux and peptic ulcer disease [1]. By inducing hypergastrinemia, PPIs have several effects on the human gastric mucosa, presenting, among many other aspects, as gastric polyp formation. Gastric polyps are classified into multiple types; benign ones are mostly represented by fundic gland polyps (FGPs) and hyperplastic polyps [2]. FGP, which can be sporadic or associated with an inherited polyposis syndrome such as familial adenomatous polyposis (FAP), are usually small-sized benign lesions, predominantly located in the fundus or body of the stomach. Microscopically, these polyps are composed of cystically dilated fundic glands [3]. Concerning hyperplastic polyps, these are also benign lesions, possibly due to PPI-induced hypergastrinemia, which causes microscopically observable foveolar epithelial hyperplasia [2].

A causal association between long-term PPI use and gastric polyps has been suggested since 1992, but the data are conflicting [4]. Moreover, few prospective studies have investigated the prevalence of gastric polyps linked to long-term use of PPIs. In Lebanon, no study has been published that highlights the link between long-term PPI use and the development of gastric polyps, including the different characteristics and risk factors that could play a role in their development. For this reason, we designed a prospective cross-sectional study conducted at two large endoscopic medical centers in Beirut, Lebanon, to primarily estimate the prevalence of gastric polyps linked to long-term PPI use. Secondary objectives include identifying the effects of sex and age group on the development of gastric polyps linked to long-term PPI use, estimating the minimal duration of daily PPI use, the dosage and the type of PPI that could cause the development of gastric polyps, and evaluating the size, location, and histological type of the gastric polyps caused by long-term PPI use.

This cross-sectional prospective study aims to estimate the prevalence of gastric polyps linked to long-term PPI use in Lebanon. Secondary objectives include identifying the effect of sex predilection and age group on the development of gastric polyps linked to long-term PPI use; estimating the minimal duration of PPI use that could cause the development of gastric polyps; estimating the dosage of PPI use necessary to cause the development of gastric polyps; identifying the type of PPI most likely linked to the development of gastric polyps; evaluating the size of gastric polyps caused by long-term PPI use; identifying the location of gastric polyps most related to long-term PPI use; and determining the histological type of gastric polyps most related to long-term PPI use.

## Materials and methods

Study population

This prospective cross-sectional study was carried out on patients presenting to the Gastroenterology Endoscopic Department at two hospital centers in Beirut: Rafic Hariri University Hospital and Zahraa Hospital University Medical Center. The patients presenting to these two centers include both inpatients and outpatients with a similar socio-economic status. Over a period of 12 months, from September 2021 to September 2022, 987 patients presenting to the endoscopic service of these two mentioned hospital centers were included in the study.

Data collection

The demographic and clinical data of the patients were all collected via a questionnaire, which included: case number; age; sex; history of smoking; history of alcohol ingestion; history of NSAID intake; history of gastric polyps; history of Helicobacter pylori; history of prior gastroscopy; history of PPI use; indication, type, dose, and duration of the PPI used; indication for this gastroscopy; presence of gastric polyp; number, size, type, location, and histology of the gastric polyp; and the result of the Helicobacter pylori test. For each patient consulting at the two endoscopic units, consent was obtained for participation in this study after explaining the aim of the study. Approximately half an hour was given for each patient to fill out the questionnaire. Special attention was given to identifying the type of PPI used by each patient by showing them a list of different types/brand names of PPI available in Lebanon, and the precise dose and duration of PPI used. The endoscopists at these two hospital centers were informed beforehand about the study, without knowing whether the patient for whom they would perform the endoscopy was taking PPIs or not. This strategy was implemented to reduce bias. Consequently, they took time to search for polyps and noted the presence of gastric polyps if found, along with their characteristics during each gastroscopy. It is important to note that the Boston Scientific endoscopic system and the BLI classification system were used to characterize gastric polyps, which were described according to the Paris classification.

Inclusion Criteria

All adult patients (over 18 years old) of both genders, whether inpatient or outpatient, presenting to the two aforementioned Gastroenterology endoscopic departments, under daily treatment with PPIs for at least one month, were included in this study.

Exclusion Criteria

Exclusion criteria for the study included patients with a personal or family history of hereditary polyposis, patients on PPI therapy for less than one month, and patients who take PPIs intermittently. Also excluded were patients who had gastric polyps and tested positive for Helicobacter pylori, those with a history of gastric polyps before PPI treatment, and patients with hypergastrinemia induced by conditions other than PPI use, such as Zollinger-Ellison syndrome, previous gastric surgery, acromegaly, pernicious anemia, and pheochromocytoma. It is important to note that gastrin levels were not measured in this study.

Statistical analysis

All analyses were carried out using the statistical software SPSS V17 for Windows, and an alpha value of 5% was established; therefore, associations with a p-value <0.05 were considered statistically significant.

## Results

Data from 987 patients were collected during the study period. After eliminating patients according to the earlier mentioned exclusion criteria (884 patients excluded), a total of 103 patients were included in this cross-sectional study. The main reasons for exclusion were patients who take PPI intermittently, followed by patients on PPI therapy for less than one month.

Prevalence

Among all the patients included in the study who had been on daily PPI for more than one month, 31 (30%) out of 103 patients were found to have gastric polyps.

Characteristics of the population

Among the 103 patients included in this cross-sectional study, the mean age was 46.48 years, and the majority were females (61.2% female vs. 38.8% male). Most were smokers (85%), non-alcoholic (79%), with no history of NSAID use (72.8%), nor a history of H. pylori infection (92.2%). The main indication for their present upper GI endoscopy was epigastric pain/dyspepsia (95%) (Table 1). The mean duration of PPI use in this study was 6.37 years. The median dosage of PPI used was 34.078 mg.

**Table 1:** Descriptive analysis of patient characteristics in the study. NSAID: Non-steroidal anti-inflammatory drug; H. pylori: Helicobacter pylori.

Individual characteristics	Category	Number of patients		Percentage %
Sex	Female	63		61.2
	Male	40		38.8
Age group	Minimum (y)	20		
	Maximum (y)	87		
	Mean (y)	46.48		
Smoker	Yes	88		85
	No	15		15
Alcohol intake	Yes	22		21
	No	81		79
History of NSAID use	Yes	28		27.2
	No	75		72.8
History of previous H. pylori	Yes	8		7.8
	No	95		92.2
Indication of gastroscopy	Pain/dyspepsia	98		95
	Reflux	5		5
Presence of gastric polyps	Yes	31		30
		Female 24	Male 7	
	No	72		70
		Female 39	Male 33	

**Figure 1:**
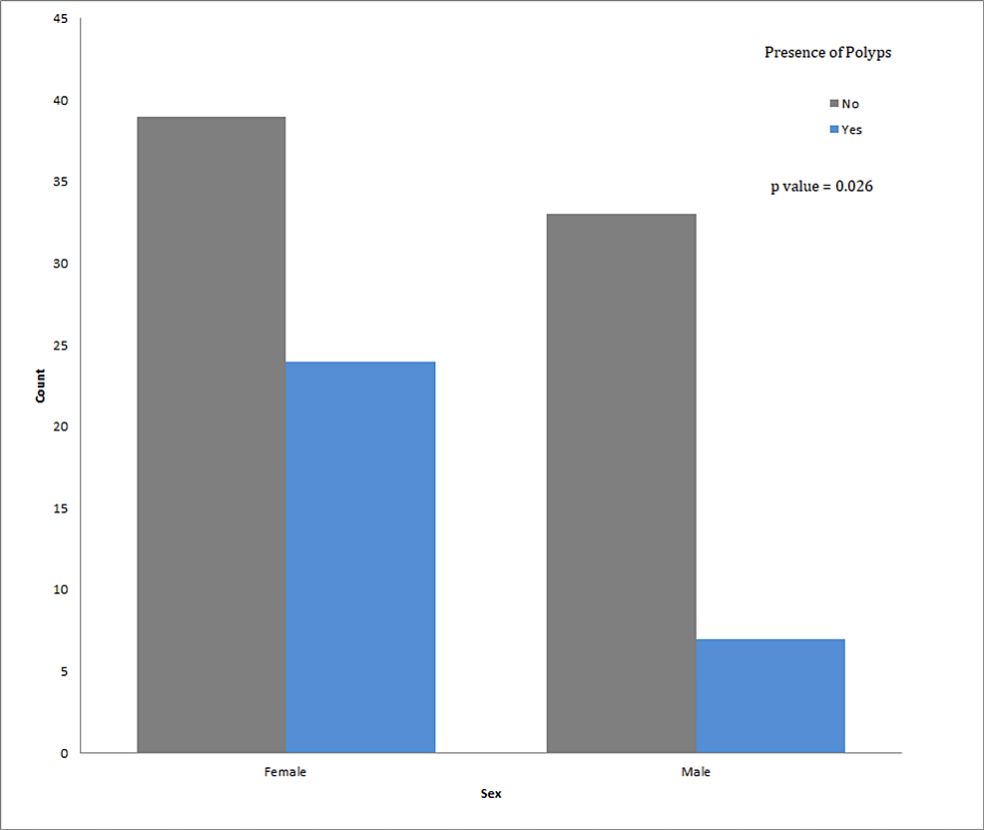
The significant correlation between sex and the presence of gastric polyps in chronic PPI users. PPI: Proton Pump Inhibitor.

To study the correlation between age and the presence of polyps, we applied Fisher’s exact test. This resulted in a p-value of 0.005, showing a significant correlation where increasing age leads to a higher risk of developing gastric polyps linked to chronic PPI use. Regarding sex, females predominated in the studied population (61.2% female vs. 38.8% male). Additionally, within the patients who presented with gastric polyps, polyps were more prevalent in the female group (77.4% female vs. 22.6% male) with an OR for gender of 2.9 for females. The Pearson Chi-Square test statistic value applied is 4.933, resulting in a p-value of 0.026 (<0.05), thus making this correlation significant (Table 2, Figure 1).

**Table 2:** Distribution of gastric polyps according to sex in patients with chronic PPI use (*p-value: 0.026). PPI: Proton Pump Inhibitor; %: Percentage.

			Presence of polyp		Total
			No	Yes	
Sex	Female*	Count	39	24	63
		% within presence of polyp	54.20%	77.40%	61.20%
	Male	Count	33	7	40
		% within presence of polyp	45.80%	22.60%	38.80%
Total		Count	72	31	103
		% within presence of polyp	100.00%	100.00%	100.00%

Correlation between duration of PPI use and presence of polyps

Patients included in this study were on daily PPI for more than one month. After collecting the different duration intervals (from several months to several years), we regrouped the patients into four categories to facilitate the statistical analysis: A total of 40/103 were on daily PPI for less than three years, 35/103 for a period between three and seven years, 17/103 for a period between 7 and 10 years, and 11/103 for more than 10 years (Table 3).

**Table 3:** Duration of PPI treatment and the presence of gastric polyps. d: Duration.

			Presence of polyp		Total
			No	Yes	
Duration of PPI use (years)	≤3	Count	39	1	40
		Percentage	54.2%	3.2%	38.8%
	3	Count	22	13	35
		Percentage	30.6%	41.9%	34.0%
	7	Count	8	9	17
		Percentage	11.1%	29.0%	16.5%
	>10	Count	3	8	11
		Percentage	4.2%	25.8%	10.7%
Total		Count	72	31	103
		Percentage	100.0%	100.0%	100.0%

**Figure 2:**
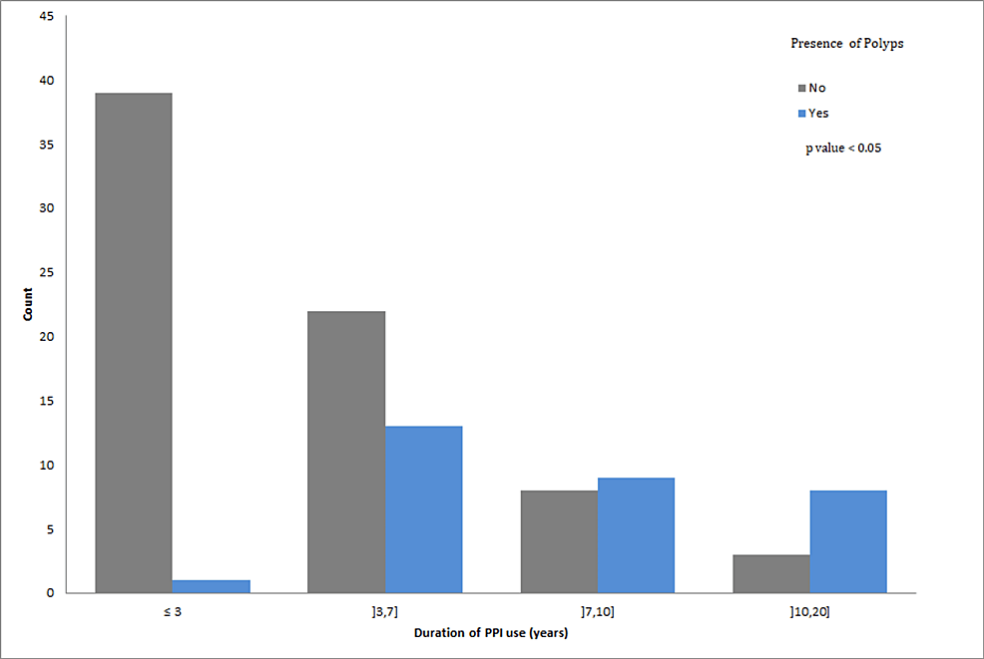
Significant correlation between the duration of PPI use and the presence of gastric polyps. PPI: Proton Pump Inhibitor.

Then, to interpret the correlation between the duration of PPI use and the presence of gastric polyps, we calculated the prevalence of polyps, which is the ratio of the number of people with polyps in each group to the total population in the same duration category (Table 4). In the group of patients who used PPI for less than 3 years, 1 out of 40 developed a gastric polyp (prevalence of 0.025), noting that this patient had been taking PPI for more than one and a half years. This prevalence increased proportionally with the increasing duration of daily PPI use: 0.37 (13/35), 0.53 (9/17), and 0.8 (8/11) for patients on daily PPI for a duration between 3 and 7 years, between 7 and 10 years, and more than 10 years, respectively. We then calculated the value of the Pearson Chi-Square test statistic, which is 29.024. This results in a negligible p-value, indicating a significant correlation between the duration of PPI use and the presence of polyps (Figure 2). We also noted that the highest number of patients presenting with polyps were in the duration frame between 3 and 7 years of PPI use.

**Table 4:** Prevalence of gastric polyps according to the duration of PPI intake (*p-value <0.05). PPI: Proton Pump Inhibitor; d: Duration.

Duration of PPI used (years)*	Prevalence of polyps
≤3	0.025
3	0.3714
7	0.53
d>10	0.8

Correlation between the dose of PPI used and the presence of polyps

Patients were divided into three categories according to the daily dosage of PPI: 27/103 were on 20 mg daily, of which 6/27 presented with a gastric polyp. 7/103 were on 30 mg daily, of which 1/7 presented with a gastric polyp, and 69/103 were on 40 mg daily, of which 24/69 presented with a gastric polyp (Table 5). When we calculated the risk for each category and applied the Pearson Chi-Square test, we found no significant correlation between the dose of daily PPI used and the presence of gastric polyps (with a p-value of 0.371, which is greater than 0.05).

**Table 5:** Presence of gastric polyps according to the dosage of PPI use (*p-value: 0.371). PPI: Proton Pump Inhibitor.

Dose of PPI (mg)	Presence of polyp		Total
	No	Yes	
20	21	6	27
30	6	1	7
40	45	24	69
Total	72	31	103

Correlation between the type of PPI used and the presence of polyps

**Figure 3:**
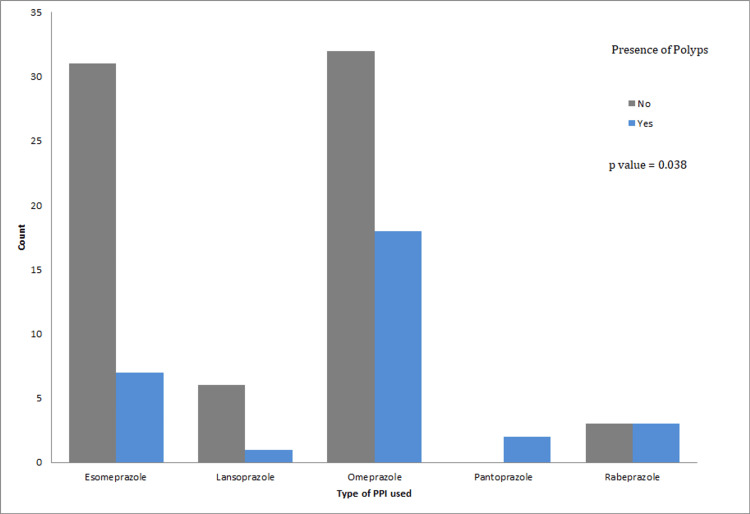
Significant correlation between the type of PPI used and the presence of gastric polyps. PPI: Proton Pump Inhibitor.

Different types of PPIs used by patients were collected. Table 6 shows the distribution of patients into each type, with the percentage of presence or absence of gastric polyps according to each category. We found that gastric polyps were present in all types of PPIs. The value of Fisher’s exact test statistic is 8.992, resulting in a p-value of 0.038. Therefore, there is a correlation between the type of PPI and the presence of polyps (Figure 3).

**Table 6:** Types of PPI used and the presence of gastric polyps. PPI: Proton Pump Inhibitor; %: Percentage.

Type of PPI			Presence of polyp		Total
			No	Yes	
	Esomeprazole	Count	31	7	38
		% within presence of polyp	43.1%	22.6%	36.9%
	Lansoprazole	Count	6	1	7
		% within presence of polyp	8.3%	3.2%	6.8%
	Omeprazole	Count	32	18	50
		% within presence of polyp	44.4%	58.1%	48.5%
	Pantoprazole	Count	0	2	2
		% within presence of polyp	0.0%	6.5%	1.9%
	Rabeprazole	Count	3	3	6
		% within presence of polyp	4.2%	9.7%	5.8%
Total		Count	72	31	103
		% within presence of polyp	100.0%	100.0%	100.0%

In order to better evaluate the association between each type of PPI used and the presence of gastric polyps, we calculated the prevalence in each group, which is the ratio between the number of patients who developed gastric polyps for every type of PPI used and the number of patients who used this type of PPI (Table 7). We found that gastric polyps were more likely to develop under Pantoprazole and least likely under Lansoprazole, with prevalences of 100% and 14%, respectively.

**Table 7:** Prevalence of gastric polyps according to the type of PPI used (*p-value: 0.038). PPI: Proton Pump Inhibitor.

Type of PPI*	Prevalence
Esomeprazole	0.184
Lansoprazole	0.142
Omeprazole	0.36
Pantoprazole	1
Rabeprazole	0.5

Correlation between the dose and duration of PPI use, with the size of gastric polyps

**Figure 4:**
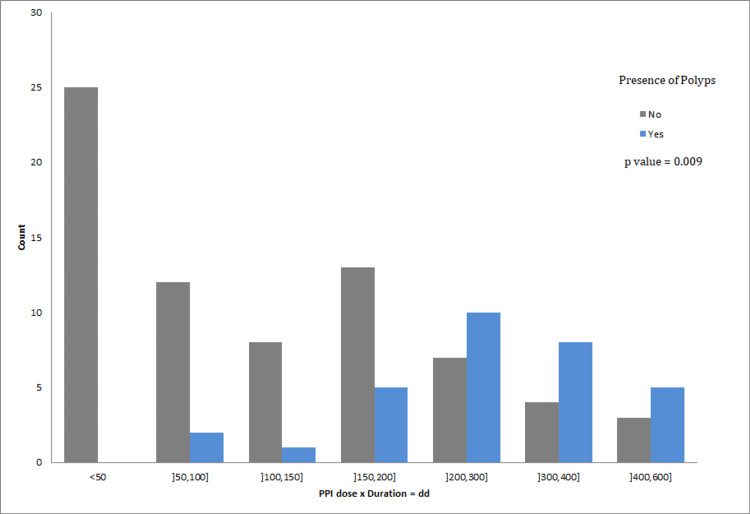
Significant correlation between the new variable 'dd' and the presence of gastric polyps linked to PPI use. PPI: Proton Pump Inhibitor; dd: Dose x duration of PPI use.

To better study the correlation between the dose and duration of PPI use and the size of the present polyps, we created a new variable, which we called “dd”, representing the product of the PPI dose and the duration of its use. We then calculated Fisher’s exact test for this new variable, with results shown in Table 8. The value of this test is 40.077, resulting in a p-value of 0.009. This indicates a correlation between the new variable “dd” and the presence of gastric polyps linked to long-term PPI use (Figure 4).

**Table 8:** Correlation between the new variable 'dd' and the presence of polyps (*p-value: 0.009). PPI: Proton Pump Inhibitor; dd: PPI dose x duration; %: Percentage.

			Presence of polyp		Total
			No	Yes	
dd	<50	Count	25	0	25
		% within presence of polyp	34.70%	0.00%	24.30%
	(50,100)	Count	12	2	14
		% within presence of polyp	16.70%	6.50%	13.60%
	(100,150)	Count	8	1	9
		% within presence of polyp	11.10%	3.20%	8.70%
	(150,200)	Count	13	5	18
		% within presence of polyp	18.10%	16.10%	17.50%
	(200,300)	Count	7	10	17
		% within presence of polyp	9.70%	32.30%	16.50%
	(300,400)	Count	4	8	12
		% within presence of polyp	5.60%	25.80%	11.70%
	(400,600)	Count	3	5	8
		% within presence of polyp	4.20%	16.10%	7.80%
Total		Count	72	31	103
		% within presence of polyp	100.00%	100.00%	100.00%

Furthermore, we calculated the correlation between this new variable “dd” and the size of polyps. The results, shown below, reveal that Fisher’s Exact Test statistic is 90.607 with a p-value of 0.013, indicating that the size of a gastric polyp is linked to PPI use and increases proportionally with the combined dose and duration of daily PPI use.

Location of gastric polyps linked to long-term PPI use

Among patients who presented with gastric polyps linked to long-term PPI use, the predominant location was the fundus (17/31, 54.83%) compared to 8/31 (25.8%) in the antrum and 4/31 (12.9%) in the body. Additionally, two patients had polyps in two locations within the stomach: one patient had polyps in both the fundus and antrum, and the other had polyps in both the fundus and the body.

We also wanted to evaluate the correlation between the duration of PPI use and the location of the present gastric polyps. We noted that these two patients with polyps in two different locations had been using PPIs for more than 10 years (Table 9). When we applied Fisher’s exact test, the results showed no significant correlation between the duration of PPI use and the location of gastric polyps, with a p-value of 0.108 (>0.05).

**Table 9:** Repartition of gastric polyps within the stomach according to the duration of PPI use (p-value: 0.108). PPI: Proton Pump Inhibitor; d: Duration.

Duration of PPI use (years)	Fundus	Body	Antrum	Fundus + Body	Fundus + Antrum	Total
d≤3	1	0	0	0	0	1
3	10	2	1	0	0	13
7	4	2	3	0	0	9
d>10	2	0	4	1	1	8
Total	17	4	8	1	1	31

Histological type of gastric polyp linked to long-term PPI use

Among patients who presented with gastric polyps linked to long-term PPI use, the most common histological type was fundic gland polyps (FGPs), comprising 67.74% (21/31), compared to 25.8% (8/31) for hyperplastic polyps and 6.4% (2/31) for inflammatory polyps.

**Figure 5:**
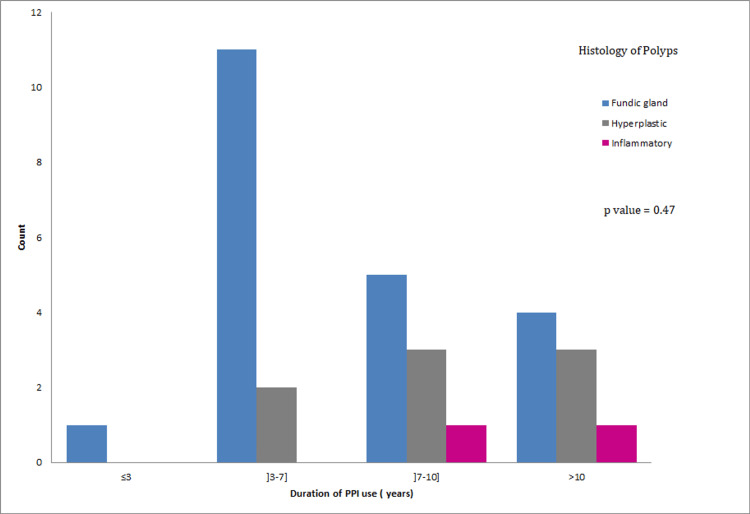
The repartition of the three histological types of gastric polyps according to the duration of PPI use. PPI: Proton Pump Inhibitor.

Furthermore, we wanted to evaluate if there is a correlation between the duration of PPI use and the histological type of the present gastric polyps (Figure 5). Table 10 shows the distribution of the three histological types of gastric polyps according to the duration of PPI use. When applying Fisher’s exact test, the result showed no significant correlation between the two, with a p-value of 0.47 (>0.05).

**Table 10:** Repartition of the three histological types of gastric polyps according to the duration of PPI use (p-value: 0.47). PPI: Proton Pump Inhibitor; d: Duration.

Duration of PPI use (years)	Histology of polyp			Total
	Fundic gland	Hyperplastic	Inflammatory	
≤3	1	0	0	1
3	11	2	0	13
7	5	3	1	9
d>10	4	3	1	8
Total	21	8	2	31

## Discussion

This conducted cross-sectional prospective study showed a prevalence of 30% of gastric polyps linked to PPI use. The most common location was the fundus, and the predominant histological type was FGPs, independent of the duration of PPI use. There was a significant correlation with the female sex with an odds ratio of 2.9. This result is in line with the recent cross-sectional study conducted by Hatano Y et al., where FGPs linked to chronic PPI use correlated with the female sex, with an odds ratio of 1.77 [5]. Concerning the duration of daily PPI use and the presence of gastric polyps, the results showed a significant correlation between them. We noticed that none of the patients who used PPI for less than two years developed gastric polyps, suggesting that a minimum duration of 24 months of daily PPI use is needed to develop gastric polyps. We identified three types of gastric polyps, similar to the findings of the retrospective study conducted by Choudhry U et al. [6]: hyperplastic, inflammatory, and FGPs, with the latter being the predominant histological type. This observation aligns with the results from the prospective study conducted by Hongo M et al. in Japan [2], and with the findings of the recent meta-analysis conducted by Martin FC et al. [4], where the majority of gastric polyps linked to daily PPI use were FGPs, and the risk of FGPs increases with the duration of PPI use. In contrast to our study, they showed a shorter duration between daily PPI use and the formation of gastric polyps where more than 12 months were sufficient to lead to FGP formation [2, 3, 4]. This slight difference could be due to the number of patients included in their studies and the risks of bias. However, other studies, such as the one conducted by Ally MR et al., showed the same findings as our results, with a critical threshold of 24 months of daily PPI duration for the development of these polyps [7]. The exact underlying pathophysiology of the formation of FGPs is still unclear, with suggestions that activating mutations of the β-catenin gene play a role in the formation of sporadic FGPs [3]. Hypotheses involve chronic PPI use, which leads to a hypergastrinemia state by neutralizing the acidic environment of the stomach. The resultant state, in part, stimulates the oxyntic mucosa, leading to parietal cell hypertrophy and hyperplasia, and increases the cytoplasmic secretory canaliculi, in other parts, resulting in parietal cell protrusions that may be the precursors to the formation of FGPs [7].

In our study, the prevalence of hyperplastic polyps linked to chronic daily PPI use was 25.8%, higher than the 8.9% found in a prospective study conducted by Hongo M et al. [2]. Additionally, we did not find in our study the higher incidence of hyperplastic polyps in H. pylori-positive patients on chronic PPI use, which has been demonstrated by Hongo M et al. [2]. In fact, in our study, all patients with H. pylori positive on biopsy did not show any gastric polyps. This calls into question the protective role of H. pylori infection, previously mentioned by some studies, in preventing the formation of FGPs.

Concerning the dosage of daily PPI, our results found no significant correlation with the presence of gastric polyps. This finding is corroborated by previous studies, such as the one conducted by Ally MR et al., which showed no significant difference in the prevalence of FGPs between once-daily versus twice-daily dosing [7]. Regarding the type of PPI used, the present study showed a prevalence of 100% with the use of pantoprazole. This likely could be due to chance, considering that only one patient used pantoprazole and had a gastric polyp. Although the statistical analysis showed a significant correlation between the type of PPI used and the formation of gastric polyps, the interpretation of these findings is difficult and questionable. This is due to the wide heterogeneity in the number of patients in each PPI category and the non-randomization of patient selection in the study, which introduces bias from patients potentially taking several types of PPI. However, despite these limitations, this cross-sectional study could serve as a starting point for future studies to better evaluate whether the type of PPI could affect the risk of polyp formation, considering that each type of PPI may be associated with a different prevalence of side effects, such as the significantly stronger association of microscopic colitis with lansoprazole compared to other types of PPI [8]. Regarding size, we considered a new variable which we called 'dd' that was significantly associated with the presence of polyps, in order to better evaluate the correlation between the amount of PPI taken (which includes the duration and the dosage of daily PPI) and the size of the resultant gastric polyp. Our findings correlated with those of the meta-analysis conducted by Martin FC et al., which demonstrated that the size of gastric polyps increases with PPI intake [4].

Furthermore, we noticed that the number of patients with positive H. pylori in our study was 39/103. After eliminating the patients with a history of previous H. pylori infection to limit the risk of bias (which is 8, of which 2 had again positive H. pylori), we estimated the prevalence of H. pylori in symptomatic patients (experiencing dyspepsia, epigastric pain) in our study to be approximately 39% (37/95) with a male predominance (22/37). This prevalence is lower than the 66.6% estimated in a study from northern Iran by Toosi SM et al. [9], but it aligns with the global prevalence of H. pylori infection of around 50% in developing countries versus 10-20% in developed countries [10]. Additionally, to better estimate the prevalence of gastric polyps linked to long-term PPI use, we excluded all patients with a history of H. pylori infection to reduce bias, given that H. pylori infection status could be an important confounding factor, as shown by several studies where the development of FGPs is known to be suppressed by H. pylori infection [2]. Moreover, we decided to eliminate all patients, if any, with both positive H. pylori and the presence of gastric polyps in this study. However, of the 39 patients with a positive H. pylori, none showed gastric polyps. As a result, we did not eliminate any additional patients.

Impact of the study

Several cases of FGPs linked to PPI use, with dysplasia or carcinoma, have been reported [11, 12]. Although there is no current data to date that proves an association between gastric cancer and PPI-induced FGPs, no case of dysplasia within the FGPs was demonstrated in our study. However, the incidence of FGPs has increased according to the wide range of chronic use of PPIs; therefore, the potential risk of dysplasia/canceration should draw attention [13]. It should be noted that the relationship between PPI and the formation of gastric polyps is reversible once PPI is discontinued [14, 15]. Considering the high number of prescriptions for PPI worldwide, and especially in Lebanon, as well as the side effects associated with this drug, including the dilemma of gastric polyps associated with long-term use of PPI and the risks of dysplasia, the present study aims to assess the prevalence of gastric polyps linked to long-term use of PPI. This study highlights the importance of limiting the prescription of PPI to globally well-defined indications and determining the various risk factors that promote the link between gastric polyps and the use of PPI, as well as the characteristics associated with these polyps.

Limitations

Several studies have shown a link between chronic use of PPI and the formation of gastric polyps, with fundic gastric polyps being predominant, but only a few of them were prospective. This is what strengthens our model. Being a prospective cross-sectional study, the endoscopists were informed about the study, so they paid closer attention to detecting the presence of any gastric polyps and their related characteristics. In addition, with the elimination of H. pylori infection and the presence of a state of hypergastrinemia or a personal/family history of gastric polyp, we limited every confounding factor that could affect the accuracy of the statistical values of our study. Furthermore, this is the first study in Lebanon, and one of the fewest, to our knowledge, to stratify the different characteristics linked to the formation of gastric polyps in a chronic PPI user in a significant statistical way.

However, our study is limited by the number of the population. This is due to the fact that the present study was conducted during a period of economic crisis in Lebanon, where hundreds of patients were financially unable to proceed to gastroscopy, or were obliged to take PPIs intermittently and even to change the type of PPI used, according to the cheapest available, all of which were factors of elimination in our model, in order to preserve the high accuracy of the statistical results. In addition, patients included in this study were supposed to be those on a daily PPI, of one type, for which no observation or randomization was done, all of which constitute a risk of bias.

## Conclusions

This cross-sectional study shows that the prevalence of gastric polyps linked to chronic daily PPI use in Lebanon was 30%. The minimal duration of daily PPI use required for the formation of gastric polyps is around 24 months. The dosage did not play a significant role in increasing this prevalence. These polyps were predominantly found in females, increased with age, were mostly of the fundic gland type, and located in the fundus. Their size was proportionally linked to the combination of both the dosage and the duration of daily PPI use.
